# Short and long-term lifestyle coaching approaches used to address diverse participant barriers to weight loss and physical activity adherence

**DOI:** 10.1186/1479-5868-11-16

**Published:** 2014-02-12

**Authors:** Elizabeth M Venditti, Judith Wylie-Rosett, Linda M Delahanty, Lisa Mele, Mary A Hoskin, Sharon L Edelstein

**Affiliations:** 1 Western Psychiatric Institute and Clinic, University of Pittsburgh Medical School, 3811 O’Hara Street, Pittsburgh, PA 15213, USA; 2 Department of Epidemiology and Population Health, Albert Einstein College of Medicine, 1300 Morris Park Avenue, Bronx, NY 10461, USA; 3 Diabetes Research Center, Massachusetts General Hospital, Harvard Medical School, 50 Staniford Street, Suite 340, Boston, MA 02114, USA; 4 George Washington University Biostatistics Center, 6110 Executive Boulevard, Suite 750, Rockville, MD 20852, USA; 5 Southwestern American Indian Center, ACKCO Inc., 1616 Indian School Road, Suite 470, Phoenix, AZ 85016, USA

**Keywords:** Lifestyle intervention, Diabetes prevention, Barriers, Behavioral approaches, Problem-solving, Toolbox strategies

## Abstract

**Background:**

Individual barriers to weight loss and physical activity goals in the Diabetes Prevention Program, a randomized trial with 3.2 years average treatment duration, have not been previously reported. Evaluating barriers and the lifestyle coaching approaches used to improve adherence in a large, diverse participant cohort can inform dissemination efforts.

**Methods:**

Lifestyle coaches documented barriers and approaches after each session (mean session attendance = 50.3 ± 21.8). Subjects were 1076 intensive lifestyle participants (mean age = 50.6 years; mean BMI = 33.9 kg/m^2^; 68% female, 48% non-Caucasian). Barriers and approaches used to improve adherence were ranked by the percentage of the cohort for whom they applied. Barrier groupings were also analyzed in relation to baseline demographic characteristics.

**Results:**

Top weight loss barriers reported were problems with self-monitoring (58%); social cues (58%); holidays (54%); low activity (48%); and internal cues (thought/mood) (44%). Top activity barriers were holidays (51%); time management (50%); internal cues (30%); illness (29%), and motivation (26%). The percentage of the cohort having any type of barrier increased over the long-term intervention period. A majority of the weight loss barriers were significantly associated with younger age, greater obesity, and non-Caucasian race/ethnicity (p-values vary). Physical activity barriers, particularly thought and mood cues, social cues and time management, physical injury or illness and access/weather, were most significantly associated with being female and obese (p < 0.001 for all). Lifestyle coaches used problem-solving with most participants (≥75% short-term; > 90% long term) and regularly reviewed self-monitoring skills. More costly approaches were used infrequently during the first 16 sessions (≤10%) but increased over 3.2 years.

**Conclusion:**

Behavioral problem solving approaches have short and long term dissemination potential for many kinds of participant barriers. Given minimal resources, increased attention to training lifestyle coaches in the consistent use of these approaches appears warranted.

## Background

The Diabetes Prevention Program (DPP) demonstrated that diabetes incidence was reduced 58% with lifestyle intervention and 31% in the metformin compared to the placebo treatment group [1]. Approximately half of the lifestyle group reached a 7% weight loss goal and three-quarters met the 150 minute weekly physical activity goal by the end of 16 sessions; 37% and 67% of the cohort remained at weight and activity goals, respectively, after an average 3.2 years. Other reports have discussed variables associated with behavioral success [2-4], the relative impact of weight loss and physical activity on diabetes incidence [5] and key intervention features [6]. Because lifestyle intervention was successful, a group-facilitated program was implemented in all treatment arms, providing a model for cost-effective diabetes prevention translation [7]. However, the kinds of barriers DPP participants faced or the individualized approaches lifestyle coaches used to facilitate adherence have not been explored. Examining these data may inform group-based training and dissemination efforts currently underway. The original DPP lifestyle intervention was highly resourced, but some of the coaching strategies may be translatable to group-facilitated approaches.

The cost-effectiveness of the original DPP treatments [8-10] has been addressed and a burgeoning dissemination literature demonstrates that standardized adaptations are feasible and effective in producing weight losses of roughly 3-7%, with decreased cardio-metabolic risk, at least in the short term [11-30]. The Centers for Disease Control (CDC) National Diabetes Prevention Program (NDPP) and others have focused on training a competent workforce to implement DPP-adapted interventions with fidelity, and build infrastructure to sustain group based diabetes prevention programs [26,31]. Similarly, the IMAGE project has established common primary prevention training standards and practice guidelines in Europe [32,33]. Nonetheless, skepticism remains regarding long-term effectiveness of behavioral interventions for maintaining population level changes in eating, activity and weight to reduce diabetes incidence [34,35]. Criticisms that such programs require significant time, costly skilled labor and additional products, or that adherence is unpredictable, have been answered in part by the early success of DPP dissemination efforts [13-30]. Nonetheless, understanding adherence barriers among a large ethnically diverse participant group, and the specific methods (referred to as “toolbox approaches”) used by lifestyle coaches have implications for translation. It is not possible to discriminate the effectiveness of single strategies in a multi-component behavioral intervention, but quantifying commonly used coaching approaches adds to our knowledge of how best to translate a known effective intervention to the community at large.

Problem-solving is central to obesity interventions [36-40]. Explicit guidance in this area distinguishes behavior modification from educational approaches or brief dietary consultation. Problem solving is a behavior change method used in conjunction with other approaches such as goal setting, self-monitoring and feedback, behavioral prompts and rehearsal, cognitive coaching, and reinforcement for goal achievement [37]. Lifestyle coaches frequently employ such techniques when interacting with participants and utilize five problem-solving steps including: [1] positive orientation; [2] problem definition/behavior chains; [3] generating alternatives; [4] setting achievable goals and [5] trial and error implementation. Despite the important role of this approach, few prospective studies have been conducted. Perri and colleagues [38-40] have demonstrated that extended programs for obese women, using problem solving for self-management, are associated with better outcomes compared to standard behavior therapies or education-only interventions. Murawski et al. [40] found that participants with ≥ 10% weight reductions demonstrated significantly greater improvement on a self-report measure of problem-solving skill than those with < 5% reductions. Problem-solving capacity has been linked to coping with chronic illness, including diabetes self-management [41-46], and interventions have targeted this specifically. The current study analyzes participant barriers and the toolbox approaches used to address a range of adherence problems in the DPP. These findings have implications for program cost and training approaches.

## Methods

As reported previously [47], 3234 participants at 27 centers (68% women, 45% from ethnic and minority groups, and 20% ≤ age 60) enrolled in DPP, were randomly assigned to either the intensive lifestyle, metformin, or placebo arm between 1996 and 1999 and followed for an average 3.2 years. The intensive lifestyle arm included 1079 participants and 1076 completed at least one intervention session. Intervention manuals and materials may be found at http://www.bsc.gwu.edu/dpp/manuals.htmlvdoc. Intervention methods pertinent to the current analyses follow.

### DPP lifestyle intervention protocol

In sessions 1–8, participants learned self-regulatory skills for goal-setting, self-monitoring of food intake, activity and body weight, managing environmental cues, energy balance, and problem-solving. In sessions 9–16, they were guided to respond to psychological (thought and mood) cues, social cues, stress, life events and other motivational challenges/barriers using these strategies. During the post-core or maintenance phase (commencing about 6 months from baseline) participants were seen at least bi-monthly, with interim phone or mail contact, to continue skill review and problem-solving as needed. Previous data shows that participants attended 23.6 ± 7.1 sessions during the first year of intervention and 12.5 ± 7.1 sessions in the second year for a total mean attendance of 50.3 ± 21.8 sessions over 3.2 years [3].

### Process evaluation

Lifestyle coaches recorded participant weight, physical activity minutes and fat and calorie intake from the previous week after each session. In addition, they identified: 1) the most critical barriers to weight loss and physical activity progress and 2) the coaching strategies (toolbox approaches) used per session.

#### Barriers

Lifestyle coaches (not the participants) coded barriers from a standardized list that was compiled, a priori, by experts (Table 1). The protocol instructed coding a barrier as present when it was discussed in the session and/or the participant’s self-monitoring, weight, or activity records reflected the problem. A barrier was coded as present as long as it appeared to be impeding forward movement. The prompt was: “If the participant is not progressing toward weight loss or physical activity goals, what is getting in the way?” Up to three weight loss barriers (from a list of 13) and up to three physical activity barriers (from a list of 12) were coded. Multiple barriers could be present over the course of treatment and the lifestyle coach named the three most important ones influencing the participant’s weight loss and activity efforts since the last contact. Barriers were defined as impediments to progress that the participant could be expected to anticipate and modify with continued training and support. If no significant challenges could be identified (e.g. progress was slow but the participant engaged in all of the expected training elements of the intervention), the lifestyle coach was instructed to report “none”.

**Table 1:** Standardized list used by DPP lifestyle coaches to report participant barriers

***Weight loss***	***Physical activity***
1. Poor/inconsistent self-monitoring	1. Poor/inconsistent self-monitoring
2. Social cues for unhealthy eating	2. Social cues for activity changed
3. Vacation, holiday, celebrations	3. Vacation, holiday, celebrations
4. Infrequent physical activity	4. Injury
5. Internal (thought and mood) cues	5. Internal (thought and mood) cues
6. Poor food shopping/food preparation skills	6. Lack of access/Safety concerns
7. Major life events	7. Major life events
8. Time management and planning	8. Time management and planning
9. Illness	9. Illness
10. Diminished motivation	10. Diminished motivation
11. Bored/dissatisfied with healthy eating	11. Aches and pains
12. Quit smoking	12. Activity restricted by doctor
13. Pregnancy	

#### Toolbox approaches

Lifestyle coaches also coded, from a standardized list (see Table 1), the behavior change strategies used to promote lifestyle progress. The prompt was: “What approaches were taken to improve or maintain weight loss (2a) or physical activity (2b)?” Essentially two levels of tool box strategies were utilized for problems with attendance, self-monitoring, and other barriers. Level 1 included no-cost behavioral methods implemented in the context of an approximate 60 minute visit along with the lesson material for that session; level 2 involved increased time, labor or additional monetary costs. Protocol training emphasized that coaches use simple, no-cost strategies before progressing to more complex, time intensive or costly approaches. Up to 3 of over 25 possible weight loss and over 25 physical activity approaches were coded for each lifestyle session including the option of “none”. Similar approaches were collapsed as summarized in Table 2a and 2b (e.g., “referral to specialist” could mean dieticians, exercise specialists, behavior specialists or other doctor). The DPP included a budget to enhance participant adherence to lifestyle intervention goals (up to $100 per participant, per year). Because it is unlikely that the U.S. health care system will support Level 2 lifestyle intervention strategies, we are particularly interested in highlighting the translation potential for Level 1 methods used. More specific information about DPP toolbox approaches may be found at http://www.bsc.gwu.edu/dpp/lifestyle/apndxg.pdf.

**Table 2:** Intervention approaches to improve weight loss and physical activity adherence (N = 1076)

	**Toolbox approaches**	**CORE (1-16)**	**POST-CORE**
**a. Weight loss approach**	***Level 1 (Standard)***		
	Problem-Solving	77%	96%
	Review Self-Monitoring Skills	49%	76%
	Recommend Increased Activity	35%	76%
	Recommend Lower Fat/Cal Goal	24%	25%
	Negotiate New Self-Monitoring Strategy	16%	47%
	Provide Healthy Recipes	14%	37%
	Develop Motivational Strategy	13%	25%
	Recommend Use of Structured Meal Plans	10%	40%
	***Level 2 (Extra Time or Added Cost)***		
	Schedule Extra Phone Call or Visit	18%	75%
	Propose Incentive Strategy or Contract**	11%	52%
	Extra Mailings; Recommend/Provide Slim Fast Shakes; Refer to Specialists; Involve Family Members; Provide Low Fat/Cal Frozen Entrees, Food Samples, Taste Testing, Cookbooks, Utensils, Loan/Buy Self Help Books, Grocery Store Visit	<10%	0-30%
**b. Physical activity approach**	***Level 1 (Standard)***		
	Problem-Solving	74%	91%
	Exercise With Participant in Session	18%	48%
	Develop Motivational Strategy (No Cost)	14%	24%
	Refer to Exercise Facility (No Cost)	10%	19%
	Refer to Exercise Specialist (No Cost)	10%	22%
	Make Plan to Find Regular Exercise Partner	9%	24%
	***Level 2 (Extra Time or Added Cost)***		
	Schedule Extra Phone Call or Visit	16%	64%
	Propose Incentive Strategy or Contract**	8%	44%
	Loaned Item to Support PA (e.g. heart rate monitor)	8%	18%
	Purchase Item to Support PA	8%	26%
	Provide Trial Health Club Membership	6%	14%
	Gave Pedometer	3%	41%
	Extra Mailings, Refer to Specialists, Involve Family Members, Loan/Buy Self-Help Books or Exercise Equipment, Register for Community Activity Event	<5%	0-30%

*Note: Intervention approaches are rank-ordered by the percentage of participants for whom they were used at least once. Lifestyle coaches recorded “no additional approach was used” during at least 1 core and 1 post-core curriculum session for nearly 100% of participants.

**Incentive strategy (Added Cost) is an approach that entails making behavior contracts with participants to set short-term (e.g. 4-6 week) measurable goals (e.g. increasing physical activity minutes by 30 minutes per week, limiting snacks to no more than 200 calories). A small reward is provided (e.g. 10$ gift certificate to sporting goods or grocery store) only if the goal is achieved.

### Statistical analysis

Descriptive analyses included rank ordering of weight loss and physical activity barriers and intervention approaches. Data were reported as the percentage of participants for whom a given barrier or approach was coded at least once. Analyses were conducted separately for the 16-session core- and later post-core period because it was hypothesized that barriers and intervention approaches might change over time. The first half and second half of the 16-session curriculum was also analyzed separately but differences were negligible, thus the first sixteen sessions remained a single unit of analysis.

Baseline demographic variables were defined at DPP study randomization. Separate cluster analyses were conducted on the barrier data for weight loss (13 variables) and physical activity (12 variables) using the VARCLUS algorithm for oblique components [48]. It has been theorized that VARCLUS is superior to orthogonal approaches when there is an assumption that all underlying factors are relatively highly correlated [49]. This method was shown to aid interpretation in a recent study analyzing the latent factor structure among multiple risk factors in the metabolic syndrome [50]. All observed barrier variables were divided into discrete (non-overlapping) subgroups that were relatively highly correlated with one another but distinct from other subgroups. The authors confirmed that the resulting groupings were clinically meaningful. Once clusters were identified, participants reported to have any one of the barriers in the cluster were considered positive for that category.

Four weight loss barrier clusters were identified initially: 1) self-monitoring, cooking and shopping, not enough physical activity, time management; 2) internal cues, bored or dissatisfied, low motivation for change; 3) social cues and context, vacations and holidays; and 4) life events and illness. Because self-monitoring is well-established as a critical behavior change target for weight loss [3,37], it was examined as the fifth stand-alone variable, independent from barrier cluster 1. For physical activity, five main barrier clusters were identified: 1) internal cues and low motivation for change; 2) self-monitoring; 3) vacations and holidays, time management, life events; 4) injury, temporary restriction, illness, and 5) no access to suitable environments for activity and weather.

Finally, Chi-squared tests were performed to examine the relationship of participants’ baseline demographic features to each of the five weight loss and physical activity barrier categories identified. When the demographic characteristic was linear in nature, as was the case for baseline age and BMI, the Mantel-Haenszel test for trend was employed. SAS (Cary NC) version 9.2 was used for all analyses [51].

## Results

At baseline, the average age of the 1076 DPP lifestyle participants was 50.6 years, mean BMI was 33.9 kg/m^2^ and the cohort was 68% female and 48% non-Caucasian. Current analyses are based on those who completed at least one session during each of the core- and the post-core intervention phases (N = 1037 or 96% of baseline sample).

### Participant barriers

**Figure 1:**
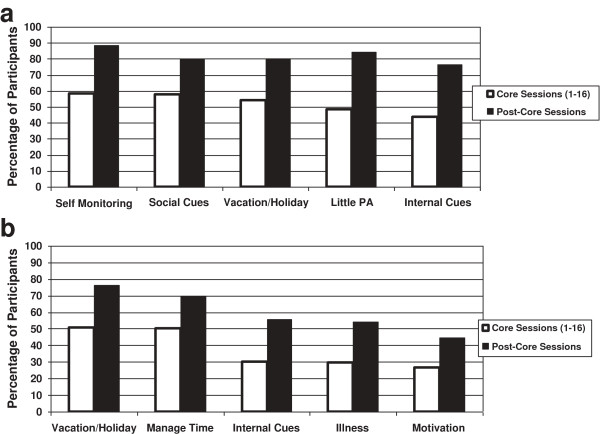
**(a) Top five weight loss barriers & (b) Top five physical activity barriers (N = 1076).** Note: Participant barriers are shown as the percentage of DPP participants for whom they were recorded at least once. Lifestyle coaches also recorded “no barrier” for nearly 100% of participants during at least 1 core and 1 post-core curriculum session.

With respect to lifestyle progress, “no barrier” was coded for 100% of DPP participants at least once and, on average, five to six times, during both phases of the lifestyle core and post-core intervention. This suggests that lifestyle coaches were not compelled to report the presence of barriers at every encounter and at least some of the time they did not observe any. When barriers were reported, the top five observed for weight loss (Figure 1a) during the first 16 sessions (based on percentage of participants for whom they were noted) included problems with self-monitoring (58%), social cues (58%), vacations and holidays (54%), too little physical activity (48%) and internal (thought/mood) cues (44%). Each barrier was reported for a larger proportion of participants (> 75%) during the post-core phase, demonstrating that with longer treatment duration lifestyle coaches observed and documented more, not fewer, barriers to progress. This is consistent with a sizable behavioral weight modification literature that indicates many, but not all, participants struggle with adherence after the first six months of intervention and that some manner of continued contact, behavioral prompts, and coaching support is required to suppress the rate of weight regain [37,38,52,53]. The most commonly reported physical activity barriers (Figure 1b) included holidays (51%), time management (50%), internal (thought/mood) cues (30%), illness (29%) and motivation (26%). Lifestyle coaches also observed an increase of up to 75% of participants, during the post-core intervention period, for the physical activity barriers of holidays and time management, and somewhat less of an increase for internal cues, illness, and motivation. Overall, physical activity barriers were coded for a smaller proportion of DPP participants compared to weight loss barriers.

#### Toolbox approaches

Table 2 shows that during both early and later intervention phases of DPP, the majority of the lifestyle cohort was successful with standard session content alone; no additional toolbox strategies were implemented. After “no approach” was excluded from these rankings, problem-solving was the dominant intervention approach used to help most participants make progress towards either the weight loss goal (2a) or the physical activity goal (2b) during both intervention phases. Other Level 1 toolbox strategies commonly used included review of basic self-management skills and recommendations to increase activity or decrease fat and calories. All toolbox strategies, including Level 2, were used with a greater proportion of participants over time. For example, to improve weight loss progress, extra phone calls or treatment sessions were scheduled with only 18% of participants during the core intervention but that number increased to three-quarters (75%) of participants during the post-core intervention, presumably as individual barriers were noted to increase. A similar trend was noted for extra phone calls or visits during the later treatment phase to promote physical activity.

However, the most costly supplemental intervention approaches (e.g. food provision, referral to specialists, exercise equipment or gym enrollment, or other material incentives) were utilized with a relative minority of participants during any phase of the DPP lifestyle intervention (< 10% during core sessions and < 30% during post-core sessions).

#### Weight loss barrier groupings and demographic characteristics

Table 3 displays the five weight loss barrier groupings, defined for sessions 1–16, in relation to baseline demographic characteristics. Several consistent associations were found. Internal cues (e.g. self-defeating thoughts and mood) affected a significantly larger proportion of women than men (p < 0.001), younger (25 to < 44 years) compared to older participants (≥ 60 years) (p < 0.05), those with high BMI (≥ 35 kg/m^2^) compared to leaner individuals (p < 0.01), non-Caucasians compared to Caucasians (p < 0.001) and single/widowed persons compared to those married/living together (p < 0.001). The same directional pattern was found for the association of life or illness events with sex, age, and BMI, (all p’s < 0.001), race/ethnicity (p < 0.01), and marital status, (p < 0.05). No sex differences were apparent for the barrier grouping of basic lifestyle skills (i.e., shopping, food preparation and meal-planning) or dietary self monitoring. However, basic skills were significantly more challenging for younger vs. older (p < 0.001), heavier vs. leaner (p < 0.05) and non-Caucasian vs. Caucasian (p < 0.001) participants as was adherence to dietary self-monitoring (age, p < 0.001, BMI, p < 0.01, and race/ethnicity, p < 0.001).

**Table 3:** Weight loss barrier categories during DPP core sessions (1-16) by baseline demographic characteristics

**Baseline demographic characteristics**			**Basic skills**	**Self- monitoring**	**Internal cues (thought/mood)**	**Life events/illness**	**Social cues**
**Feature**	**Level**	**All**	**Yes**	**Yes**	**Yes**	**Yes**	**Yes**
		**N =**	**%**	**%**	**%**	**%**	**%**
Sex	Male	343	56	54	**40**^**c**^	**22**^**c**^	69
	Female	733	61	60	**52**	**34**	71
Age* (years)	25 to <44	356	**66**^**c**^	**64**^**b**^	**52**^**a**^	**38**^**c**^	73
	45 to 59	487	**58**	**56**	**47**	**29**	70
	60 and older	233	**50**	**54**	**43**	**21**	70
BMI* (kg/m^2^)	< 30	357	**55**^**a**^	**53**^**b**^	**43**^**b**^	**26**^**c**^	69
	30 – 34.9	334	**59**	**58**	**46**	**27**	70
	35+	385	**63**	**63**	**55**	**38**	73
Race/Ethnicity	White	578	**50**^**c**^	**50**^**c**^	**43**^**c**^	**26**^**b**^	**65**^**c**^
	African-American	203	**70**	**74**	**58**	**35**	**77**
	Hispanic	178	**67**	**58**	**46**	**39**	**75**
	American Indian	60	**78**	**82**	**62**	**35**	**78**
	Asian	57	**68**	**61**	**54**	**26**	**82**
Work Status	Working	783	60	59	49	**32**^**b**^	**73**^**a**^
	Retired	148	50	55	40	**20**	**67**
	Other	145	61	57	50	**31**	**62**
Marital Status	Single/Widowed	370	60	62	**56**^**c**^	**35**^**a**^	73
	Married/Living Together	706	59	56	**44**	**28**	70
Education	≤ 12^th^ Grade	279	63	**63**^**a**^	47	31	74
	13 or more	797	58	**56**	48	30	70
Household Income	< 35 K	347	62	**64**^**b**^	51	32	70
	35 to 75 K	422	59	**58**	49	31	74
	75 K or more	224	57	**52**	44	26	70

Weight loss barrier clusters that differ significantly by demographic characteristics are highlighted in **bold font** (with a superscript notation indicating the p-value level).

^*^The Mantel-Haenszel test for trend was used for statistical analyses with the Age and BMI variables; the Chi-squared test was used for all other variables.

^a^p <0.05.

^b^p <0.01.

^c^p <0.001.

We examined whether increased barriers among younger compared to older individuals might be related to work status and associated time demands. The aforementioned barrier categories (internal cues, basic skills, and self-monitoring) were not shown to be different for working compared to retired persons. However, retired compared to employed participants were observed to have significantly fewer life events or illness challenges (p < 0.01), or problem social cues (p < 0.05), as weight goal barriers. The impact of chronological age and developmental life stage on adherence to lifestyle interventions may be difficult to distinguish completely. Finally, individuals with less household income (p < 0.01) or fewer years of education (p < 0.05) had significantly more difficulty with dietary self-monitoring.

To further examine a possible interaction between race/ethnicity and either household income or education, unstratified and stratified odds ratio analyses were conducted. Race/ethnicity were collapsed into binary categories of Caucasian vs. non-Caucasian because of the small number of participants in several of the subgroups. No interaction was found for either this binary variable or household income or level of education on any of the five weight loss barrier categories defined. Thus it appears that non-Caucasian groups experienced more barriers to weight loss compared to Caucasians, independent of their socioeconomic status.

#### Demographic characteristics associated with physical activity barrier clusters

Table 4 presents results on the relationship of five physical activity barrier groupings to baseline demographic features as reported for sessions 1–16. These associations did not simply mirror those found for weight loss. Lifestyle coaches rarely coded self-monitoring (< 10%) as a barrier to achievement of physical activity goals suggesting that participants either did better at tracking their activity, or tracking was not related to goal achievement in the same way as it was for weight loss.

**Table 4:** Physical activity barrier categories during DPP core sessions 1-16 by baseline demographic characteristics

**Baseline demographic characteristics**			**Internal cues (Thought/mood)**	**Self-monitoring**	**Social cues & time management**	**Physical events (Injury/illness)**	**Access/weather**
**Feature**	**Level**	**All**	**Yes**	**Yes**	**Yes**	**Yes**	**Yes**
		**N =**	**%**	**%**	**%**	**%**	**%**
Sex	Male	343	**32**^**c**^	6	**61**^**c**^	**33 **^**c**^	**24 **^**b**^
	Female	733	**44**	8	**75**	**48**	**32**
Age* (years)	25 to <44	356	**43**^**a**^	9	**77**^**c**^	46	30
	45 to 59	487	**41**	7	**72**	43	28
	60 and older	233	**33**	8	**56**	38	30
BMI* (kg/m^2^)	< 30	357	**31**^**c**^	7	**61 **^**c**^	**36 **^**c**^	**22 **^**c**^
	30 – 34.9	334	**37**	8	**70**	**42**	**27**
	35+	385	**51**	8	**79**	**50**	**37**
Race/ Ethnicity	White	578	**36**^**b**^	6	67	43	**26 **^**b**^
	African-American	203	**47**	10	72	47	**37**
	Hispanic	178	**37**	8	77	38	**26**
	American Indian	60	**58**	4	72	43	**25**
	Asian	57	**42**	13	75	44	**43**
Work status	Working	783	**42**^**b**^	8	**74**^**c**^	**45 **^**a**^	30
	Retired	148	**28**	6	**53**	**33**	26
	Other	145	**42**	6	**66**	**43**	28
Marital status	Single/Widowed	370	**46**^**b**^	9	68	**49**^**b**^	**36 **^**c**^
	Married/Living Together	706	**37**	7	72	**40**	**26**
Education	≤ 12^th^ Grade	279	39	8	67	40	**34**^**a**^
	13 or more	797	41	7	71	44	**28**
Household income	< 35 K	347	44	8	68	42	**33**^**a**^
	35 to 75 K	422	40	7	71	42	**34**
	75 K or more	224	35	7	72	45	**21**

Physical activity barrier clusters that differ significantly by demographic characteristics are highlighted in **bold font** (with a superscript notation indicating the p-value level).

^*^The Mantel-Haenszel test for trend was used in statistical analyses of Age and BMI; the Chi-squared test was used for all other variables.

^a^p <0.05.

^b^p <0.01.

^c^p <0.001.

Demographic differences were noted for several other barriers to physical activity progress, most notably internal (thought and mood) cues. Self-defeating thoughts were a bigger problem for women than men (p < 0.001), younger compared to older persons (p < 0.05), those with higher vs. lower BMI (p < 0.001), non-Caucasians, working persons and those living alone compared their counterparts (all p’s < 0.01) indicating that the psychological aspects of adherence to activity interventions may be worthy of closer investigation. Access and weather barriers affected women more than men (p < 0.01), heavier compared to leaner individuals (p < 0.001), non-Caucasians compared to Caucasians (p < 0.01), and those living alone compared to couples (p < 0.001). Access/weather was the only barrier that distinguished those with lower levels of education and income (p < 0.05) from more highly educated and less economically challenged participants. With respect to problem social cues and time management, women, more obese individuals, and those who worked had more difficulty than their counterparts (all p’s < 0.001). These three subgroups also had significantly more problems with physical injury or illness (p < 0.001, p < 0.001, p < 0.05, respectively).

Unstratified and stratified statistical analyses were conducted to see if there were significant interactions between race/ethnicity and either household income or education. None were found for any physical activity barrier grouping.

## Discussion

This report is the first to present process data regarding the coach-participant interaction during the successful DPP lifestyle intervention. A main finding of this clinical trial was that a goal-based behavior change intervention was more efficacious than drug or placebo treatment in delaying diabetes onset over 3.2 years [1,2]. Although an ethnically and racially diverse group of lifestyle participants succeeded, on average, the current analysis showed that they had to manage a wide variety of problems to do so. For dissemination it is helpful to examine the major barriers and the different types of lifestyle coaching approaches used to improve short and long term adherence. First, the diet and activity barriers and their demographic variability are characterized followed by a discussion of the most common lifestyle coaching approaches used.

This analysis showed that self-defeating thoughts and mood, problem social cues, and disrupted physical activity routines were more common for women than men, younger compared to older persons for some barriers, and working compared to retired persons for others. These findings extend previous data showing that DPP lifestyle participants over the age of 60 had better session attendance, turned in more food records and demonstrated more favorable long term weight loss, physical activity participation, [3] diabetes delay and other biometric outcomes [54]. One implication is that in DPP translation, barriers (or the perception of barriers) do appear related to longer term adherence and outcomes and should be addressed proactively. Another implication is that individuals over aged 60 represent a particularly “ready” subgroup for translational programs because their barriers (or perceptions of barriers) are fewer. Future studies should evaluate the cost-effectiveness of delivering lifestyle interventions to older adults whose disease risks and costs are otherwise expected to accelerate sharply.

Another implication for future research is that younger and middle-aged individuals, particularly women, appear to need amplified social support, to address common barriers including access to interventions that fit more seamlessly into the context of their daily routines. Research by Wing and Jeffery [55] and others [56] has demonstrated the benefits of recruiting participants with friends to increase social support for weight maintenance. Targeting naturally occurring social groups such as friends, co-workers, or those with common life circumstances (e.g. high risk mothers with preschoolers) may be a fruitful avenue for diabetes prevention translation. Face to face interaction has consistently been shown to be most effective for weight loss and maintenance [52,53], however telephonic and web-based approaches are increasingly being utilized as a cost-effective means to extend the reach and scope of intervention support [16,24,25,52,53].

In addition, the data suggest that a significantly larger proportion of non-Caucasians compared to Caucasians (roughly 10-20% more) were found to have multiple barriers, independent of socioeconomic status. Lower household income and less education was significantly associated with less frequent dietary self-monitoring. Self-monitoring of physical activity did not appear to be a problem for most DPP participants, but access to places to exercise or weather-related challenges were more commonly reported for racial and ethnic minorities compared to Caucasians. Because dietary self-monitoring and feedback is highly correlated with weight loss success [3,37,57], we conclude that a more accessible array of dietary self-monitoring tools is needed. Mobile applications (e.g. smart phones), especially those that offer real-time feedback, have been shown to enhance self-monitoring adherence [58] but this may not be accessible for some population subgroups. Prior research has examined flexible, alternate forms of dietary self-monitoring for those who do not adhere to traditional methods (e.g. picture-based checklists of commonly eaten foods and portion sizes) [59] but more studies are needed.

A second major finding of the current investigation is that problem-solving was the dominant short and long term coaching approach for the full range of barriers with the majority of participants. This type of approach was used with most but not all participants during the first 16 sessions of lifestyle intervention; subsequently it essentially became the basis for the coach-participant interaction. As barriers increased over the course of treatment, lifestyle coaches also turned to some more costly methods (e.g., those with low translation potential). The implication for dissemination research is that effective curriculum delivery must go well beyond didactic teaching in helping participants develop more autonomy for anticipating and responding to personal barriers and lapses. Previous obesity intervention research does not provide a unified prescription on how best to achieve this but several studies have emphasized the importance of addressing the self-defeating thoughts often associated with behavioral avoidance and relapse [40,60-64]. The IMAGE toolkit approach in Europe has also strongly emphasized the consistent use of behavior change processes that include self-monitoring and feedback, problem solving for relapse prevention, and seeking community-based social support [32,33]. We conclude that future intervention design and training of lifestyle coaches would do well to increase time spent on the practice and facilitation of problem-solving approaches. Review of dietary self-monitoring skills was the second most common coaching approach used during the short and long term intervention. Another translational consideration, therefore, is that one-on-one diary review and feedback is very time consuming for lifestyle coaches. Novel use of trained lay health coaches (e.g. alone or in conjunction with a dietician) or peer group interaction to facilitate self-monitoring review and feedback, or other digital-interactive methods may accomplish similar ends and deserve further study.

Contrary to what has been assumed regarding the DPP intervention [32], monetary based approaches (e.g. rewards for behavior change, gym memberships) were utilized for fewer than 10% of DPP participants during the first 16 sessions. Research findings have been mixed on the utility of such incentives in promoting health behavior change [65]; our results indicate they were not central to the success of the intervention. However, added-cost toolbox approaches did increase to up to 75% of participants (e.g., staff-time due to increased number of phone calls or sessions) as barriers became more evident. It is clear that many participants will benefit from ongoing behavioral counseling assistance beyond the initial six months of intervention. How best to address this need for continued primary prevention contact in the current health care environment is a critical empirical and policy question.

There are several limitations to these analyses. One is that the data was exclusively reported by the lifestyle coaches and no corollary measures were obtained from the participants, thus there is risk of a systematic reporting bias. Documentation of the treatment approaches (toolbox strategies) used was more objective because the actual methods were targeted in session and reported immediately afterwards. Another limitation is that data on the participants’ baseline or post-intervention problem-solving skills was not collected; such measures should be incorporated into future translational studies.

## Conclusion

Most Diabetes Prevention Program participants faced multiple internal, social and environmental barriers to lifestyle behavior change and all of these were observed to increase over the course of long term intervention. Session for session, repetitive problem-solving and review of self-monitoring skills were the most common lifestyle coaching approaches utilized, not costly incentives. Behavioral problem solving approaches have long term dissemination potential for many kinds of participant barriers. Given minimal resources, training lifestyle coaches to facilitate these approaches in a highly skillful manner appears warranted.

## Competing interests

All listed authors receive NIH support. In addition, Ms. Delahanty has a financial interest in Omada Health, a company that develops online behavior-change programs, with a focus on diabetes. These interests were reviewed and are managed by Massachusetts General Hospital and Partners HealthCare in accordance with their conflict of interest policies.

## Authors’ contributions

EMV, JW, LD and MH are members of the Lifestyle Advisory Group, which designed the intervention used in this study and these individuals all participated in the conceptual design of the current process analysis. SE participated in the original design and assessment protocol of the RCT and LM designed and conducted most of the statistical analysis for this report. EMV was responsible for drafting and revising the manuscript and all authors read and approved several drafts of the final manuscript.

## Supplementary Material

Additional file 1 DPP Research Group Investigators (to Aug 2002). Click here for file
